# Supporting parenting to address social inequalities in health: a synthesis of systematic reviews

**DOI:** 10.1186/s12889-018-5915-6

**Published:** 2018-08-31

**Authors:** Annabelle Pierron, Laurence Fond-Harmant, Anne Laurent, François Alla

**Affiliations:** 1 Université de Lorraine, EA4360 APEMAC, Vandoeuvre-lès-Nancy, France; 2 Luxembourg Institute of Health, Department of Population Health, 1 A-B Rue Thomas Edison, Strassen, 1445 Luxembourg; 3 Société Française de Santé Publique, 1 rue de la forêt, Laxou, 54520 France; 4 CHU INSERM. Bordeaux Population Health Research Center. UMR 1219 CIC-EC 1401, Université de Bordeaux, Bordeaux, France

**Keywords:** Social determinants of health, Disparity, Perinatology, Parenting, Health promotion, Healthcare disparities, Health status disparities

## Abstract

**Background:**

In 2009, the World Health Organization’s Commission on Social Determinants of Health set out its recommendations for action, which included establishing equity from early childhood onwards by enabling all children and their mothers to benefit from a comprehensive package of quality programmes. In order to address social inequalities in health, it is recommended that action be taken from early childhood, and actions providing support for parenting are an effective lever in this respect.

The aim of this review of systematic reviews is to analyse, on the one hand, the components and characteristics of effective interventions in parenting support and, on the other, the extent to which the reviews took into account social inequalities in health.

**Methods:**

A total of 796 reviews were selected from peer-reviewed journals published between 2009 and 2016 in French or English. Of these, 21 reviews responding to the AMSTAR and selected ROBIS criteria were retained. These were analysed in relation to the consideration they gave to social inequalities in health according to PRISMA-equity.

**Results:**

The reviews confirmed that parenting support programmes improved infants’ sleep, increased mothers’ self-esteem and reduced mothers’ anger, anxiety and stress levels. The mainly authors noted that the contexts in which the interventions had taken place were described either scantly or not at all, making it difficult to evaluate them.

Only half of the reviews had addressed the question of social inequalities in health. In particular, there had been little research conducted on the relational aspect and the social link.

**Conclusion:**

In terms of addressing social inequalities in perinatal health, the approach remains both modest and reductive. Understanding how, for whom and in what conditions interventions operate is one way of optimising their results. Further research is needed to study the interactions between the interventions and their contexts.

## Background

The social determinants of health are one of the principal causes of health inequalities, that is the unjust and sizeable discrepancies recorded between social or geographical groups [1–7]. In 2009, the World Health Organization’s (WHO) Commission on Social Determinants of Health set out its recommendations for action, which included establishing equity from early childhood onwards by enabling all children and their mothers to benefit from a comprehensive package of quality programmes [8].

Early childhood is a key period in the genesis and reproduction of Social Inequalities in Health (SIH). Epigenetic studies and life course epidemiology confirm the link between life circumstances in early childhood and health in adulthood [9–13]. Social inequalities have an effect on health, most notably by creating biological modifications throughout the life course. In particular, development is negatively influenced by antenatal and neonatal stress [14–20].

Clearly, the perinatal period is particularly sensitive. Promoting the health of pregnant women and new mothers is therefore essential, and parenting support is one of the principal strategies that can really help [13, 21–23].

Many publications show that parenting support is an effective lever in promoting health in mothers and their newborns, and they are the subject of many reviews. However, have these studies taken into account SIH? The aim of this review of systematic reviews is to analyse, on the one hand, the components and characteristics of effective interventions in parenting support and, on the other, the extent to which the reviews have taken into account SIH.

## Methods

### Study procedure

The screening, eligibility and inclusion stages were conducted and presented according to the Preferred Reporting Items for Systematic Reviews and Meta-Analyses model (PRISMA) [24, 25].

The study population was pregnant women, parents of newborns and newborns (from birth to 3 years old, preschool). The interventions involved promoting perinatal health, and more specifically, programmes and schemes offering parenting support – from the standpoint of obstetrics, paediatrics, psychology, sociology, education and public health, in the broad sense of the term.

The comparators were not relevant to this study. All the results (whether favourable or not) were collated and evaluated according to their effects on SIH, their psychosocial effects and their effects on perceived health. Intervention durations were taken into consideration.

A systematic search was conducted using Cochrane, PubMed and PsycINFO, which are the principal scientific databases in the field of health. The keywords referenced in the MeSH (Medical Subject Heading) were based on perinatal health promotion, SIH, the social determinants of health, parenting support and health programmes relating to parenting support (Table 1). Keywords were selected in collaboration with Céline Aubert, documentalist at the Faculté de Médecine in Nancy.

**Table 1:** Keywords used in the literature search

Concepts	Mots-clés du MeSH^a^	Synonymes^b^	MeSH anglais^c^	Synonymes en anglais
promotion de la santé périnatale	promotion de la santé périnatologie	promotion^d^ santé périnatalité médecine périnatale	health promotion perinatalogy	promotion^d^of health health promotion^d^
promotion de la santé	promotion de la santé		health promotion	promotion of health
lutte contre les inégalités de santé	disparités d’accès aux soins	autorestriction devant les zoins inégalité^d^ devant les soins difficulté^d^ d’accès aux soin^d^	healthcare disparit^d^	health care disparit^d^ healthcare inequalit^d^
inégalités sociales de santé	disparités d’accès aux soins disparités de l’état de santé facteurs socioéconomiques	inégalités devant les soins inégalités de l’état de santé inégalités	health care disparit^d^ health status disparities socioeconomic factors	healthcare inequalit^d^ healthcare disparit^d^ health status disparity inequalities inequlity
déterminants sociaux de la santé	déterminant social de la santé		social determinants of health	health social determinants
programme de santé relatif à l’accompagnement à la parentalité	pratiques éducatives parentales politique de santé	éducation par les parent^d^ comportement^d^ parenta^d^ éducation^d^ parentale^d^ fonction^d^ parentale^d^ politique^d^ de santé publique politique en matière de santé	parenting health policy	parenthood health polic^d^

^a^MeSH: medical subject heading

^b^synonymes: mots-clés apparaissant dans l’arborescence Mesh mais non retenus

^c^MeSH anglais: résultats produits par le CISMEF (catalogue des index des sites médicaux de langue française) à partir des mots MeSH français

^d^troncature ie développement limité

The search formulas combined the following terms: ((parenting OR parenthood) AND (“support”)), ((parenting OR parenthood) AND (“health promotion”)), ((children) AND “health promotion” AND parent*) OR ((parenting OR parenthood) AND (“health promotion”)), ((children) AND “health promotion” AND parent*) OR ((parenting OR parenthood) AND (“equity”)), ((parenting AND parenthood) AND (inequit*)), ((parenting AND parenthood) AND “health inequit*”), (parenting OR parenthood) AND (social*determinant*)) and (parenting OR parenthood) AND(disparit*)).

Each of the standardised search formulas was reproduced for all the databases, based on titles, abstracts and keywords, for journals published in French or English between January 2009 and September 2016.

The research protocol was written but not published. It is available on request from the authors.

### Selection of articles

The documents retained were all narrative and systematic reviews published in French or English in peer-reviewed journals between 2009 and 2016. The year 2009 was chosen as the start date because it was the year the WHO published its report from the Commission on Social Determinants of Health [8].

Interventions relating to prematurity, a specific pathology or to those concerning school-age children were excluded, as were reviews of reviews. Duplicate articles were also removed**.** The reason for each individual rejection was given in a note. A pilot study was carried out in May 2016 using a sample of the articles in order to validate the protocol (relevance of the search formula, selection criteria, evaluative consistency of the different team members).

The full texts of the articles selected were subject to a methodical, standardised interpretation responding to each of the AMSTAR (Assessing the Methodological Quality of Systematic Reviews) items [26]. The reviews that did not meet these criteria of methodological quality were excluded.

The selected reviews were all included whether or not their results were favourable so as to exclude the possibility of any bias resulting from selective publication of results. The grey literature and the recommendations published by France’s Haute Autorité de Santé and the UK’s National Institute for Health and Clinical Excellence were subject to an exploratory study but were not retained for this review since they did not correspond to the study aims. Table 2 shows the PICOTS criteria for the methodological search.

**Table 2:**
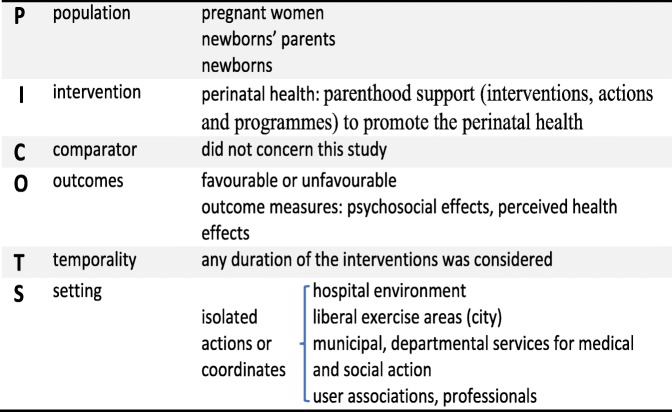
PICOTS criteria

### Literature analysis

The whole process was carried out by one person under the supervision of three public health and social sciences experts, who all agreed on the interpretation. Differences of opinion were discussed until a consensus was reached, and these were all documented.

The systematic reviews included in this review were analysed in two stages based on the full texts alone, in other words without referring to the original articles collated by the systematic reviews. Firstly, the data describing the characteristics of the interventions and programmes were compiled in a summary table. Secondly, the reviews were analysed for the consideration they gave to SIH using the PRISMA-equity tool [27]. This tool is an extension of the PRISMA guidelines and was developed by the Campbell and Cochrane Equity Methods Group. Its aim is to take into account the notion of equity in reviews. Each of the elements of the systematic reviews (i.e. the title, abstract, introduction, method, results and discussion) was broken down and analysed to determine how the authors addressed equity.

**Fig. 1:**
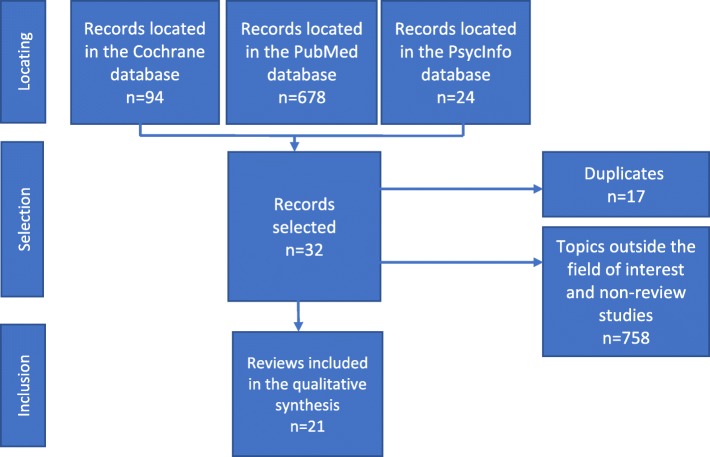
Flow diagram showing the stages of article selection

A total of 796 publications were obtained from the literature search. Once the articles that were duplicated across the databases had been taken out and those about prematurity, maternal and infantile pathologies and children aged over three had been excluded, only 32 articles remained. The next stage allowed us to exclude articles that did not meet the AMSTAR criteria (for assessing methodological quality) and the ROBIS criteria (for assessing risk of bias) (Table 3). Finally, following a detailed analysis, 21 articles were included in the corpus (Fig. 1).

**Table 3:** Evaluation of the methodological quality of the included reviews according to the AMSTAR criteria and an adaptation of the ROBIS* criteria [26, 58]

References	A priori search plan	Review adheres to pre-defined objectives and eligibility criteria (ROBIS *)	All pre-defined analyses reported or departures explained (ROBIS*)	Selection of studies, data extraction ≥2 people	Exhaustive literature search	Nature of publication/inclusion criteria	List of (included and excluded) studies provided	Indication of study characteristics	Evaluated and recorded scientific quality of included studies	Scientific quality of included studies/formulation of conclusions	Methods for combining results from suitable studies	Evaluation of probability of publication bias^a^	Declaration of conflicts of interest
Miller S, Maguire L, Macdonald K. Home-based child development interventions for preschool children from socially disadvantaged families. Cochrane systematic reviews. Dec 2011.	yes	yes	yes	yes	yes	yes	yes	yes	yes	yes	yes	yes	none declared
Barlow J, Smailagic N, Huband N, Roloff V, Bennett C. Group-based parent training programmes for improving parental psychosocial health. Cochrane systematic reviews. May 2014.	yes	yes	yes	yes	yes	yes	yes	yes	yes	yes	yes	yes	none declared
Barlow J, Smailagic N, Bennett C, Huband N, Jones H, Coren E. Individual and group based parenting programmes for improving psychosocial outcomes for teenage parents and their children. Cochrane Database of Systematic Reviews 2011, Issue 3.	yes	yes	yes	yes	yes	yes	yes	yes	yes	yes	yes	yes	none declared
Barlow J, Bergman H, Kornør H, Wei Y, Bennett C. Group-based parent training programmes for improving emotional and behavioural adjustment in young children. Cochrane Database of Systematic Reviews 2016.	yes	yes	yes	yes	yes	yes	yes	yes	yes	yes	yes	yes	none declared
Bryanton J, Beck CT, Montelpare W. Postnatal parental education for optimizing infant general health and parent-infant relationships. Cochrane Database of Systematic Reviews 2013, Issue 11.	yes	yes	yes	yes	yes	yes	yes	yes	yes	yes	yes	yes	none declared
Bennett C, Underdown A, Barlow J. Massage for promoting mental and physical health in typically developing infants under the age of six months. Cochrane Database of Systematic Reviews 2013, Issue 4.	yes	yes	yes	yes	yes	yes	yes	yes	yes	yes	yes	yes	none declared
Tiitinen S, Homanen R, Lindfors P, Ruusuvuori J. Approaches used in investigating family support in transition to parenthood. Health Promot Int. 2014 Sep;29(3):518–27.	yes	yes	yes	yes	yes	inadequate description of method	included yes	yes	yes	yes	yes	yes	not mentioned
Lakshman R, Ogilvie D, Ong KK. Mothers’ experiences of bottle-feeding: a systematic review of qualitative and quantitative studies. Arch Dis Child. 2009 Aug;94(8).	yes	yes	yes	not known	yes	yes	included yes	yes	yes, summary table	yes	yes, narrative method	not mentioned	not mentioned
Entsieh A, Hallström I. First-time parents’ prenatal needs for early parenthood preparation: a systematic review and meta-synthesis of qualitative literature. Midwifery. 2016 Aug;39:1–11.	yes	yes	yes	not known	yes	yes	included yes	yes	yes, reference PRISMA criteria	yes, reference PRISMA criteria	yes	not mentioned	none declared
Welsh J, Strazdins L, Ford L, Friel S, O’Rourke K, Carbone S, Carlon L. Promoting equity in the mental wellbeing of children and young people: a scoping review. Health Promot Int. 2015 Sep;30 Suppl 2:ii36–76.	yes	yes	yes	not known	yes	yes	included yes	yes	yes	yes	yes	not mentioned	project supported by the Australian Research Council
Panter-Brick C, Burgess A, Eggerman M, McAllister F, Pruett K, Leckman J. Practitioner review: engaging fathers – recommendations for a game change in parenting interventions based on a systematic review of the global evidence. J Child Psychol Psychiatry. 2014 Nov; 55(11): 1187–1212.	yes	yes	yes	yes	yes	yes	included yes	yes	yes, reference PRISMA and where, when, how analysis	yes, reference PRISMA and where, when, how analysis	yes	not mentioned	not mentioned
Poole MK, Seal DW, Taylor CA. A systematic review of universal campaigns targeting child physical abuse prevention. Health Educ Res. 2014 Jun;29(3):388–432.	yes	yes	yes	yes	discussed	yes	included yes	yes	yes	yes	yes, narrative method according to topic + summary tables	yes	none declared
Mcdaid D, Park AL. Investing in mental health and well-being: findings from the DataPrev project. Health Promot Int. 2011 Dec;26 Suppl 1	yes	yes	yes	not known	yes	yes	included yes	yes	yes	yes	yes, narrative method according to topic + summary tables	not mentioned	study supported by the European Parliament research programme
Perry M, Becerra F, Kavanagh J, Serre A, Vargas E, Becerril V. Community-based interventions for improving maternal health and for reducing maternal health inequalities in high-income countries: a systematic map of research. Global Health. 2015 Jul 1;10:63.	yes	yes	yes	yes	yes	yes	included yes	yes	yes	yes	yes	not mentioned	MASCOT supported by the European Commission’s Seventh Framework Programme
Morrison J, Pikhart H, Ruiz M, Goldblatt P. Systematic review of parenting interventions in European countries aiming to reduce social inequalities in children’s health and development. BMC Public Health. 2014 Oct 6;14:1040.	yes	yes	yes	assumed because references to PRISMA, but not found	yes	yes	included yes	yes	yes	yes	yes	yes	none declared
Piotrowski CC, Talavera GA, Mayer JA. Healthy steps: a systematic review of a preventive practice-based model of pediatric care. J Dev Behav Pediatr. 2009 Feb;30(1):91–103.	yes	yes	yes	not known	yes	yes	included yes	yes	yes	yes	yes	yes	not mentioned
Van Vuuren CL, Reijneveld SA, Van der Wal MF, Verhoeff AP. Neighborhood socioeconomic deprivation characteristics in child (0–18 years) health studies: a review. Health & Place, Vol 29, Sep, 2014 pp. 34–42.	yes	yes	yes	yes	yes	yes	included yes	yes	yes	yes	yes	not mentioned	not mentioned
Gilmer C, Buchan JL, Letourneau N, Bennett CT, Shanker SG, Fenwick A, Smith-Chant B. Parent education interventions designed to support the transition to parenthood: a realist review. International Journal of Nursing Studies, Vol 59, Jul, 2016 pp. 118–133.	yes	yes	yes	yes	yes	yes	included yes	yes	yes	yes	yes	not mentioned	none declared
Geens N, Vandenbroeck M. The (ab)sense of a concept of social support in parenting research: a social work perspective. Child & Family Social Work, 19: 491–500. 2014.	yes	yes	yes, but not clearly	not known	yes	yes	included yes	yes	no	yes	yes, thematic analysis	not mentioned	not mentioned
Spiteri G, Xuereb RB, Carrick-Sen D, Kaner E, Martin CR. Preparation for parenthood: a concept analysis. Journal of Reproductive and Infant Psychology, Vol 32(2), Mar, 2014 pp. 148–165.	yes	yes	yes	yes	yes	yes	included yes	yes, in the anlaysis	yes	no	yes, written record	not mentioned	not mentioned
Halford W, Kim Petch J. Couple psychoeducation for new parents: observed and potential effects on parenting. Clinical Child and Family Psychology Review, Vol 13(2), Jun, 2010 pp. 164–180.	no	not clearly	yes, but not clearly	not known	not known	not known	included yes	yes, in the anlaysis	yes	yes	yes, thematic analysis, summary tables and written record	not mentioned	not mentioned

^a^tendency to publish experiments that obtained positive results to the detriment of those that obtained negative results

All the documents constituting the corpus were read twice. In cases of uncertainty, a second expert was consulted. In cases of disagreement between the first two experts, a third was consulted.

AP, FA, LFH carried out the documentary research protocol for the selection. For the analysis, AP read all the manuscripts twice, FA validated and, in cases of uncertainty, LFH had the final say. AL and LFH reviewed the manuscript.

## Results

### Summary of evidence-based knowledge

The data produced by the review were recorded in a summary table showing the type of intervention studied in each systematic review, the review’s aims, and the levels of evidence such as they were analysed and described by the authors and the principal characteristics of the programmes [37–41] (Table 4).

**Table 4:** Synopsis of reviews relating to parenting support in the perinatal period

country where study was conducted (if multiple: country of first author)	references	strategy or intervention	strategy or intervention	review’s aim	principal results to be noted on the problem or its determinants	level of evidence	examples of validated programmes	characteristics of validated programmes
UK	Miller S. Maguire L.K. Macdonald. Home-based child development interventions for preschool children from socially disadvantaged families. Cochrane systematic reviews. Dec 2011.	home-based programmes promoting children’s cognitive and socio-emotional development	socially disadvantaged families, preschool children	to know the effects of these programmes	future studies should endeavour to better document and describe their methodological processes	no evidence of effectiveness because of a lack of documentation of the methodological processes [old studies *n* = 7, between 1973 and 1993; participants *n* = 723]	_	_
US, AUSTRALIA, UK, GERMANY, JAPAN, NETH, NZ	Barlow J. Smailagic N. Huband N. Roloff V. Bennett C.Group-based parent training programmes for improving parental psychosocial health. Cochrane systematic reviews.May 2014.	parenting support to improve the psychosocial health of parents	all parents and children	to determine whether the programmes were effective in improving psychosocial wellbeing	short-term improvement (< 6 months) in parents’ health (depression, anxiety, stress, anger, guilt, self-confidence)	significant results [studies, *n* = 48; participants *n* = 4937]	Parent Management Training, Triple P, Behavioural-Based Parenting Programme, Prevention Programme for Externalising Problem, Community Outreach Parent Empowerment, Maternal Stress Coping Group Programme, Incredible Years	behavioural and /or cognitivesupport: regular group therapy, telephone support
KOREA, JAPAN, SWI, NETH, US	Barlow J, Smailagic N, Bennett C, Huband N, Jones H, Coren E. Individual and group based parenting programmes for improving psychosocial outcomes for teenage parents and their children. Cochrane Database of Systematic Reviews 2011, Issue 3.	individual and group parental support programmes to improve psychosocial results for parents and their children	adolescent parents under the age of 20	to evaluate the effectiveness of these programmes	short- and long-term improvement in parent-child interactions. Improvement in psychosocial health: reduction in anxiety and depression, increase in self-esteem.	lack of methodological rigour and lack of a sufficiently large sample to compile enough data to produce sufficient statistical power [studies *n* = 8, participants *n* = 513]	short videos of exchanges with parent, therapy groups, education in the form of parent-child interactive simulation	behavioural support and / or improving knowledge
US, UK, CAN, AUSTRALIA, MEX, PERU	Barlow J, Bergman H, Kornør H, Wei Y, Bennett C. Group-based parent training programmes for improving emotional and behavioural adjustment in young children. Cochrane Database of Systematic Reviews 2016.	group training programmes for mothers to improve the emotional and behavioural adjustment of children	parents of children under 3	to evaluate the effectiveness of these programmes	reduction in stress and anger	moderate short-term evidence of emotional and behavioural adjustment. Insufficient data to prove the effectiveness of primary prevention and provide long-term results [studies *n* = 24; participants *n* = 3161]	1–2-3 Magic video, group therapy on parent-child interaction, interventions targeting verbal and corporal punishment, Triple P	behavioural and / or cognitive support, meetings between parents or between professionals and parents over the course of several weeks
US, NEPAL, BRAZIL, UK, CAN, AUSTRALIA	Bryanton J, Beck CT, Montelpare W. Postnatal parental education for optimizing infant general health and parent-infant relationships. Cochrane Database of Systematic Reviews 2013, Issue 11.	postnatal parental education to optimise the general health of infants and parent-infant relationships	parent groups or individual meetings, from birth to two months	to evaluate the effectiveness of these programmes	improvement in infant’s sleep, maternal knoweldge and infant’s security. Improvement in mother-father-child interaction. Improvement in infant’s language development.	evaluation and / or larger sample required to be able to draw conclusions [trials *n* = 15; mothers *n* = 2922 and fathers *n* = 402]	evaluation and / or larger sample required to be able to draw conclusions	education on the infant’s behaviour
UK, US, CAN, KOREA, ISRAEL, INDIA, TURKEY, IRAN	Bennett C, Underdown A, Barlow J. Massage for promoting mental and physical health in typically developing infants under the age of six months. Cochrane Database of Systematic Reviews 2013, Issue 4.	massage aimed at promoting the mental and physical health of infants	typically developing children under the age of six months	to evaluate the effectiveness of these programmes	future research studies will have to examine the beneficial effects of infant massage in populations that present higher risks (for example: socially disadvantaged parent-child duos)	vast majority of the studies were of poor methodological quality [studies *n* = 24]	_	_
Finnish authors, mainly English and American studies	Tiitinen S. Homanen R. Lindfors P. Ruusuvuori J. Approaches used in investigating family support in transition to parenthood. Health Promot Int. 2014 Sep;29(3):518–27.	review describing the research methods used to study parenting support interventions	mainly parents in maternity units (post-partum stay)	to know the priority research areas, methods used and groups targeted	the fathers’ issues, rarely studied separately from those of the mothers. The studies targeting parents’ needs focused mainly on socioeconomically disadvantaged populations. Very few longitudinal studies (with interviews at different stages of the pregnancy or after the birth)	articles *n* = 98 published before 2009, mainly English and American (bias linked to a consideration of epidemiological risk and the British and American health systems). Majority RCTs, with no intervention process evaluation approach (why the interventions are effective or ineffective).	_	map of recent research on support for parents in maternity units focusing on three key areas: the professionals’ representations of the families’ needs; effectiveness of the interventions; processes of care in maternity units
UK, US	Lakshman R. Ogilvie. Ong KK. Mothers’ experiences of bottle-feeding: a systematic review of qualitative and quantitative studies. Arch Dis Child. 2009 Aug;94(8).	mothers’ experiences of giving their babies formula milk	mothers bottle-feeding their newborns	to understand how the decision to use substitute milk is made	through lack of information and support, the mothers experience negative feelings (anger, guilt, worry, uncertainty)	proven evidence [studies: qualitative *n* = 6 and quantitative *n* = 17; participants *n* = 13,263]	not applicable	not applicable
UK, SWE, IRE, AUSTRALIA, SING, TURK	Entsieh A, Hallström I. First-time parents’ prenatal needs for early parenthood preparation-A systematic review and meta-synthesis of qualitative literature. Midwifery. 2016 Aug;39:1–11.	meta-analysis of the specific needs of parents preparing for parenthood	parents during the antenatal period	to contribute to existing knowledge on the specific needs of parents preparing for early parenthood	parents should have the opportunity to actively participate in the interventions, which should begin early prenatal and continue into postnatal. Introduction of family education meetings early in the postnatal period	research method based on the PRISMA guidelines. Qualitative approach [studies *n* = 12, participants *n* = 916]	family education courses for adults with participatory learning strategies. The parents call for discussions with their peers.	not applicable
AUSTRALIA	Welsh J, Strazdins L, Ford L, Friel S, O’Rourke K, Carbone S, Carlon L. Promoting equity in the mental wellbeing of children and young people: a scoping review. Health Promot Int. 2015 Sep;30 Suppl 2:ii36–76.	interventions aimed at promoting mental wellbeing and reducing inequalities	children and young people living in high-income countries [US, UK, AUSTRALIA and others]	to identify best practice according to population strata plus gaps in the evidence base in order to put forward recommendations	possibility of using a proportionate universalism approach centred on the question of equity in the promotion of mental wellbeing	encouraging proven evidence based on the interventions	the interventions were either universal or specifically targeted at children from disadvantaged families; no interventions involving social gradients	encouraging evidence that interventions and education in the family setting have succeeded in supporting positive parenting, both universally and for disadvantaged groups
“worldwide”: mainly US	Panter-Brick C, Burgess A, Eggerman M, McAllister F, Pruett K, Leckman J. Practitioner Review: Engaging fathers – recommendations for a game change in parenting interventions based on a systematic review of the global evidence. J Child Psychol Psychiatry. 2014 Nov; 55(11): 1187–1212.	interventions relating to parenting and fathers: evaluation of the implicit and explicit biases in current research approaches, interventions and policies	interventions targeting couples or fathers	to determine how fathers currently participate in parenting-related interventions and what improvements can be made to encourage their participation	the main obstacles to paternal engagement in parental education programmes were cultural, institutional, professional and operational in nature and were linked to the programme’s content, resources and policy considerations at the conception and implementation stages	proven evidence [publications *n* = 199]	the father’s investment in the role of parent impacts the mother and child	fathers have minimal involvement in programmes and interventions
US, UK, AUSTRALIA	Poole MK, Seal DW, Taylor CA. A systematic review of universal campaigns targeting child physical abuse prevention. Health Educ Res. 2014 Jun;29(3):388–432.	prevention of the physical abuse of children	universal campaigns, general population	to evaluate the effectiveness of the interventions and improve future prevention campaigns	recommandations: increase social support for parents, increase knowledge through the use of positive parenting techniques, better knowledge of a child’s physiological development	promising. Lack of evaluation of the programmes targeting general population [articles *n* = 17, for interventions *n* = 15]	Triple P	televised programmes. Internet sites
mainly US and UK	Mcdaid D, Park AL. Investing in mental health and well-being: findings from the DataPrev project. Health Promot Int. 2011 Dec;26 Suppl 1:i108–39.	mental health and wellbeing: the economic arguments	early years, parents and older people	to identify economic evaluations comparing the effectiveness and costs of interventions promoting mental health	low cost; major benefit for mothers and babies	proven evidence for parental support interventions	nurse-led actions: advice and education for mother and child	home-visit programmes for parents, or physiological education on babies’ sleep
US, AUSTRALIA, UK,CAN	Perry M, Becerra F, Kavanagh J, Serre A, Vargas E, Becerril V. Community-based interventions for improving maternal health and for reducing maternal health inequalities in high-income countries: a systematic map of research. Global Health. 2015 Jul 1;10:63.	map of community health interventions: reducing inequalities in maternal health	high-income countries. MASCOT project (Multilateral Association for Studying health inequalities and enhancing north–south and south-south COoperaTion)	to systematically identify the literature on community-based maternal health interventions and to describe the subjects that they tackle and the nature of the interventions in relation to inequalities in maternal health	the role of information networks and advertising is a pertinent but as yet unexplored research topic. This project opens the way for studies on the effectiveness and possibility of transferring interventions	further research needed to analyse and compare the effectiveness of the interventions mapped in this study [publications *n* = 119 between 2000 and 2013]	the interventions concerned breastfeeding, postnatal depression, support and capacity building in the parental role, prenatal preparation for the birth, birth planning	the interventions took place mainly in the home setting. The health professionals were the principal operators in these interventions
UK, IRE	Morrison J, Pikhart H, Ruiz M, Goldblatt P. Systematic review of parenting interventions in European countries aiming to reduce social inequalities in children’s health and development. BMC Public Health. 2014 Oct 6;14:1040.	early-childhood interventions that have reduced inequalities in children’s health and development	Europe. In reality, the interventions studied were conducted in the UK and Ireland	to identify early-childhood interventions that have reduced inequalities in children’s health and development	a more exhaustive evaluation of interventions must be carried out in a larger range of countries than just the UK and Ireland.	proven evidence plus other studies requiring a more in-depth evaluation [interventions *n* = 23]	the interventions in the review aimed to improve parenting skills and housing conditions. The study also looked at psychological therapies	higher level of evidence shown when there was a combination of educational workshops and programmes for parents and children that began during early pregnancy and included home visits from specialist personnel
authors: CAN; interventions: US	Piotrowski CC, Talavera GA, Mayer JA. Healthy Steps: a systematic review of a preventive practice-based model of pediatric care. J Dev Behav Pediatr. 2009 Feb;30(1):91–103.	“Healthy Steps for Young Children”, national programme aimed at providing parents with educational information and advice and allowing early screening	United States. Parents of newborns and their children	to systematically evaluate and summarise the “Healthy Steps” programme for young children	nurses, educators and social workers were trained for this programme on the subjects of parenting, child development and the importance of relationships and team building	proven evidence. Prospective study conducted over 3 years (cohort of newborns *n* = 5000) [articles *n* = 13]	more optimal vaccination coverage and care trajectory compared to control group	the state awarded a certification to the university hospitals, clinics and federally qualified health centres that organised six home visits during the children’s first three years. A dedicated phone line enabled parents to connect with the programme specialists.
authors: NETH; studies: NETH, CAN, US	Van Vuuren C. L. Reijneveld S.A. Van der Wal M. F. Verhoeff A.P. Neighborhood socioeconomic deprivation characteristics in child (0–18 years) health studies: A review. Health & Place, Vol 29, Sep, 2014 pp. 34–42.	list of the characteristics of disadvantage used in research examining the link between environment and children’s health	studies on the residents of socioeconomically disadvantaged neighbourhoods	to study the socioeconomic characteristics of disadvantage used in research studies on children’s health	major problem in conclusion: no evidence-based frames of reference for the socioeconomic characteristics used in the study protocols	studies *n* = 19	not applicable	major problem: the data used were those available and not those based on health. Income, employment and education were the most frequently used characteristics. Problem: no consensus on the best measures to use
US, UK, CAN and others	Gilmer C. Buchan J. L. Letourneau N. Bennett C. T. Shanker S.G.; Fenwick A. Smith-Chant B. Parent education interventions designed to support the transition to parenthood: A realist review. International Journal of Nursing Studies, Vol 59, Jul, 2016 pp. 118–133.	realist review of access to parenting interventions	pregnant women and parents of children under 1	to identify potentially effective health education interventions during the transition to parenthood period	a priori assumption of lack of parental information. No evaluation of pre-intervention “knowledge”. Hypothesis was an assumed lack of parenting skills.	no convincing evidence that a single educational programme was universally effective. Level of evidence from the studies difficult to evaluate because of a lack of description of the intervention’s conception, content, implementation or result (particularly attrition). No study evaluated the impact on the child [articles *n* = 72]	programmes were most effective when parents were already involved. A male facilitator increased the ongoing participation of the fathers. Parents followed the programmes more when they responded to their immediate issues (approach to childbirth, infant’s sleep)	mainly a series of lessons (generally between 5 and 8) covering a wide range of subjects. Frequently information brochures. Few programmes aimed at fathers.
US, CAN, UK, AUSTRALIA	Geens N. Vandenbroeck M. The (ab)sense of a concept of social support in parenting research: a social work perspective. Child & Family Social Work, 19: 491–500. 2014.	social support in parenting: the “(ab)sense” of a concept	sociology articles. No focus on perinatal period.	to explore how social support can be conceptualised through social work publications	the relational aspect is barely developed in research into social support linked to children’s education. Social support is a way of preventing or reducing the harmful risks of depression, stress, anxiety and other mental illnesses. It is also a way of increasing the feeling of control.	proven evidence [articles *n* = 28]	social support was principally studied in relation to parents’ health. Social relationships were highly pertinent for families but were given little consideration by social services	research into social links and social capital cannot exclude a recognition of inequality: the quantity and quality of resources and the way in which they are used can vary enormously according to socioeconomic class
MALTA, UK	Spiteri G. Xuereb R.B. Carrick-Sen D. Kaner E. Martin C.R. Preparation for parenthood: a concept analysis. Journal of Reproductive and Infant Psychology, Vol 32(2), Mar, 2014 pp. 148–165.	preparing for parenthood: an analysis of the concept	mothers and fathers	to analyse the concept of preparing for parenthood (discourse analysis and theoretical foundations)	parenting is unique to each individual, affected by cultural and societal expectations as well as lifestyle. Parental stress disrupts parent-child interactions, increases a child’s aggression and hyperactivity levels and generates an increased number of situations of negligence or abuse	limited evidence: few elements define the concept of preparing for parenthood	_	_
AUSTRALIA	Halford W. Kim Petch J. Couple Psychoeducation for New Parents: Observed and Potential Effects on Parenting. Clinical Child and Family Psychology Review, Vol 13(2), Jun, 2010 pp. 164–180.	critical analysis of the links between the couple’s relationship and their parenting	parent couples	to evaluate whether the couple’s relationship has a major influence on their parenting	psychoeducation has considerable potential in terms of improving a couple’s adjustment to parenthood and increasing their awareness and receptiveness in regard to their newborn	proven evidence [trials *n* = 11]	the couple’s relationship influences their parenting behaviours	_

### Characteristics of the reviews

#### Disciplines of the journals publishing the reviews

The articles were drawn from journals in a variety of disciplines. A third (*n* = 7) of the publications came from public health journals. Cochrane published six reviews on the topic. The remaining publications came from journals in the field of child psychology (*n* = 3), paediatrics (*n* = 2), midwifery (*n* = 1), social work (*n* = 1) and nursing care research (*n* = 1).

#### Authors’ countries of affiliation in which the studies were conducted

The reviews were mainly carried out by American and British authors and generally concerned interventions in the United States and the United Kingdom. There was only brief reference made to other European countries in eight of the reviews. These comprised a Finnish review, which listed only two European programmes in the 98 studies they examined [28], a Dutch review, which compared the Netherlands to the United States and Canada [29], two Cochrane reviews mentioning some studies conducted in Germany and the Netherlands [21, 30], two reviews that referenced Ireland [31, 32], one review mentioning interventions conducted in Switzerland [31] and another that compared Sweden with the United Kingdom [32].

#### Aim of the reviews

All but three systematic reviews aimed to assess the effectiveness of the programmes by evaluating their impact over a relatively short period on the mothers’ and infants’ wellbeing, the mothers’ mental health and the mothers’ adjustments to the behaviour of their babies and very young children. In three of the systematic reviews, the aim was to analyse the methods and concepts used in research relating to parenting support. One of these listed the main research areas, the methods used and the groups targeted [28]. The second studied the socioeconomic disadvantage characteristics used in research studies [29], and the third analysed the concept of preparing for parenthood [33].

#### Principal programmes studied

Generally, the reviews focused on the practices of the professionals; and the programmes they studied mainly involved improving parents’ knowledge and skills. Three reviews looked at parental needs. One of these was a meta-analysis focusing specifically on preparation for parenthood [31]. The second examined the needs of fathers [34], and the third looked at the needs of mothers feeding their children with substitute milk [35].

### Principal characteristics of the interventions

#### Recipient populations

The reviews focused on different periods and populations:

- a specific stage in the parents’ history: pregnancy (*n* = 3), the first week after giving birth (*n* = 1)
- a broad timeframe from birth to three years (from birth to two months (*n* = 1), up to six months (*n* = 2), up to one year (*n* = 1), up to three years (*n* = 5)
- socially disadvantaged families (*n* = 2)
- teenage parents (*n* = 1)
- fathers only [34] (all the others concerned mothers, parents or the couple as an entity)
- the general population through an analysis of prevention campaigns [36].

#### Main results and conclusions

All the reviews provided either convincing or promising data about the positive impact on mothers’ and children’s wellbeing of the support programmes and actions at and following birth. The programmes were found to increase the mothers’ self-esteem, reduce their anger, anxiety and stress levels, improve the infants’ sleep and promote the infant’s language development.

#### Three distinct levels of evidence

In accordance with the authors’ evaluations, as described and synthesised in their reviews, we grouped the evidence into three categories (this was chosen with reference to similar studies [37–41]):

1. proven evidence (*n* = 10): this corresponded to interventions for which the review authors concluded that the evidence was strong and unequivocal**.** The majority of the reviews was made up of interventions whose evidence was proven.
2. promising (*n* = 5): this concerned intervention results based on either insufficient statistical power (linked to the fact that the samples were too small) or the fact that it was impossible to draw long-term conclusions from the results [21, 30, 36, 42].
3. lack of evidence (*n* = 3): according to the review authors, it was difficult to evaluate the quality of evidence in the studies they analysed because either the methodological rigour was considered poor [43], it could not be objectivised [44] or there was no description of the intervention’s content, implementation or results [45].

It should be noted that the two systematic reviews on research methods and the review that sought to define the concept of preparing for parenthood escaped this categorisation.

##### The most effective programmes

Behavioural and cognitive support through therapy groups and telephone support significantly improved parents’ psychosocial health [21]. Psychoeducation showed considerable potential for improving a couple’s adjustment to parenthood and their awareness of and receptiveness to their newborn [46].

The most effective programmes were those begun before the birth and those in which the parents were able to actively participate [31].

Some of the reviews evaluated interventions promoting the mental health of parents during the first few years. They concluded that, for only a small financial outlay, the benefits for the mothers and babies were substantial [47].

##### The promising programmes

One review that was carried out on eight studies concluded that parental support programmes improved parents’ psychosocial health and promoted parent–child interactions. However, a lack of statistical power precluded any formal conclusion on teenage parents [30]. Based on an analysis of 48 studies in 2014, the same lead author produced proven evidence from among the general population. She concluded that group training programmes for mothers aimed at improving their children’s emotional and behavioural adjustment led to a short-term reduction in stress and anger, but she added that the data was not conclusive on the long-term effects [48].

Postnatal parental education programmes seeking to optimise infants’ health and parent–child relationships resulted in an improvement in sleep, in the mothers’ knowledge and in the infants’ sense of security. However, larger samples would have allowed a more formal conclusion to be drawn [23].

##### The programmes that did not prove their effectiveness

The programmes carried out in the home setting to promote socially disadvantaged families’ cognitive and socio-emotional development showed questionable results [44, 49]. Massage programmes aimed at boosting the mental and physical health of infants did not succeed in proving they were effective [43].

#### Two key areas for improvement in parenting support programmes

##### Strengthen the quality of interventions

On the whole, the parental support actions viewed parents as an entity and did not differentiate the fathers’ issues from those of the mothers [43]. Moreover, there were cultural and institutional obstacles to the fathers’ involvement in parental education programmes, and these need to be taken into account at the design and implementation stages of programmes aimed at this particular group [34].

Parents should have the opportunity to actively participate in interventions, and these should begin in the early prenatal stage and continue into the postnatal period. Furthermore, health promotion stakeholders seem to assume a lack of parental skills. Parental knowledge should be evaluated prior to the intervention [45]. Finally, the value of peer groups is an under-researched area [30] .

##### Develop complementary research

Longitudinal studies were extremely under-represented. Nevertheless**,** a paper included in the Cochrane database claimed that long-term programmes focusing on teenage parents had long-term positive influences on parent–child relationships [30]. Longitudinal studies could be conducted by carrying out interviews at different points during pregnancy or after the birth [28].

A number of authors stated that the level of evidence of an intervention’s effectiveness was difficult to evaluate due to a lack of any description given of the intervention’s design, content, implementation or results [43–45]. Understanding how and in what context interventions were carried out appears to be essential for the evaluative framework [28].

There are few elements that define the concept of preparing for parenthood [33].

The roles of advertising and information networks could be pertinent research topics, but they remain unexplored as yet [42].

There has been little development of the relational aspect in the research on social support [42, 50].

A systematic review of European studies conducted between 1999 and 2013 concluded that an evaluation of interventions must be carried out in a wider range of countries than just the United Kingdom. For example, France, Germany and Italy were not represented at all in the literature [32], thus creating an “Anglo-American bias” [28].

### Consideration given to SIH in the reviews

To analyse the consideration given to SIH in efficacy analyses**,** each of the elements of the reviews (i.e. the title, abstract, introduction, method, results and discussion) were broken down and analysed. The results of this analysis are summarised in Table 5.

**Table 5:** Level of consideration given to social inequalities in health, summary adapted from PRISMA-equity

references	title	abstract			introduction	methods	results	discussion	
	notion of equity identified in the title	research question linked to equity in health	results from the analyses of equity in health	description corresponds to disadvantaged population	description of hypotheses that show intervention can impact equity	study design linked to equity	results and conclusions correspond to equity	applicability to socioeconomic gradients and cultural plurality	implications for research, practice and policy corresponding to equity
Miller S. Maguire L.K. Macdonald. Home-based child development interventions for preschool children from socially disadvantaged families. Cochrane systematic reviews.Dec 2011.	socially disadvantged families	research focused specifically on socially disadvantaged families	it is described: no negative impact on the control group	no, not in the abstract	mention of social disadvantage	RCT “habitual residence” family vs socially disadvantaged family (i.e. living in poverty, lone parent, ethnic minority)	presentation of interventions by type of social disadvantage targeted. No description of results concerning the possible impact on reducing SIH.	interventions targeted disadvantaged populations, stratified according to three categories (poverty, lone parenthood, ethnic minority)	yes, mention of the intervention’s positive impact in helping “to eradicate inequalities”
Barlow J. Smailagic N. Huband N. Roloff V. Bennett C.Group-based parent training programmes for improving parental psychosocial health. Cochrane systematic reviews.May 2014.	no	no	no	no	no	no	differentiation of fathers-mothers-parents results. No negative impact from the interventions.	father-mother distinction	no
Barlow J, Smailagic N, Bennett C, Huband N, Jones H, Coren E. Individual and group based parenting programmes for improving psychosocial outcomes for teenage parents and their children. Cochrane Database of Systematic Reviews 2011, Issue 3.	targeted at teenage mothers	no, the research focused specifically on an a priori disadvantaged population	no	the target population was considered a priori to be disadvantaged	mention of the population as often coming from very disadvatantaged backgrounds, liable to suffer from a lack of social support	no	no	brief comment: “caution should therefore be exercised before the findings are generalised to other social and cultural contexts”. Mention of the value of peer groups.	no
Barlow J, Bergman H, Kornør H, Wei Y, Bennett C. Group-based parent training programmes for improving emotional and behavioural adjustment in young children. Cochrane Database of Systematic Reviews August 2016.	no	no	no	no	the authors wrote: “parental psychosocial adversity and disruptions in the parent-child relationship are significant risk factors for infant emotional, behavioural, eating and sleeping disorders”	no	no	no	no
Bryanton J, Beck CT, Montelpare W. Postnatal parental education for optimizing infant general health and parent-infant relationships. Cochrane Database of Systematic Reviews 2013, Issue 11.	no	no	no	no	no	no	no	no	no
Bennett C, Underdown A, Barlow J. Massage for promoting mental and physical health in typically developing infants under the age of six months. Cochrane Database of Systematic Reviews 2013, Issue 4.	no	no	no	no	no	no	no	no	research perspectives proposing to focus on the impact of baby massage in groups of socially disadvantaged parents
Tiitinen S. Homanen R. Lindfors P. Ruusuvuori J. Approaches used in investigating family support in transition to parenthood. Health Promot Int. 2014 Sep;29(3):518–27.	no	no	no	the authors wrote: “A bias towards the perspectives of risk groups […] was detected”	improve available knoweldge on the psychological and social factors that enhance family relations and protect children’s development	no	no	studies carried out on “at risk” populations. Little attention given over to universal services	need to examine the interventions’ mechanisms to understand which interventions are (in)effective and in what contexts
Lakshman R. Ogilvie. Ong KK. Mothers’ experiences of bottle-feeding: a systematic review of qualitative and quantitative studies. Arch Dis Child. 2009 Aug;94(8).	no	no	no	no	no	no	no	no	no
Entsieh A, Hallström I. First-time parents’ prenatal needs for early parenthood preparation: a systematic review and meta-synthesis of qualitative literature. Midwifery. 2016 Aug;39:1–11.	no	no	no	no	no	no	no	no	no
Welsh J, Strazdins L, Ford L, Friel S, O’Rourke K, Carbone S, Carlon L. Promoting equity in the mental wellbeing of children and young people: a scoping review. Health Promot Int. 2015 Sep;30 Suppl 2:ii36–76.	yes: “equity”	“equitably”, “inequities”, “social gradients”, “proportionate universalism”			“life course trajectories of social and emotional prosperity, or social and emotional disadvantage”, “Interventions need to be ‘matched’ to [...] contexts”, “social determinants shape [...] inequities”, “interventions which are universal but proportionate [...] for addressing inequities”	yes	yes	yes	yes, mention of the risk of increased inequalities with certain interventions
Panter-Brick C, Burgess A, Eggerman M, McAllister F, Pruett K, Leckman J. Practitioner Review: Engaging fathers – recommendations for a game change in parenting interventions based on a systematic review of the global evidence. J Child Psychol Psychiatry. 2014 Nov; 55(11): 1187–1212.	no	no	notion of equity to be developed	no	no	no	no	yes, mother-father parity	notion of equity to be developed
Poole MK, Seal DW, Taylor CA. A systematic review of universal campaigns targeting child physical abuse prevention. Health Educ Res. 2014 Jun;29(3):388–432.	no	no	no	no	no	no	no	no	no
Mcdaid D, Park AL. Investing in mental health and well-being: findings from the DataPrev project. Health Promot Int. 2011 Dec;26 Suppl 1:i108–39.	no	no	no	no	no	no	no	no	to consider a number of levels of approach with some interventions targeted at the general population and some solely at high-risk groups
Perry M, Becerra F, Kavanagh J, Serre A, Vargas E, Becerril V. Community-based interventions for improving maternal health and for reducing maternal health inequalities in high-income countries: a systematic map of research. Global Health. 2015 Jul 1;10:63.	yes: “maternal health inequalities”	“community-based interventions”	no	no	yes	yes	yes	yes	yes
Morrison J, Pikhart H, Ruiz M, Goldblatt P. Systematic review of parenting interventions in European countries aiming to reduce social inequalities in children’s health and development. BMC Public Health. 2014 Oct 6;14:1040.	yes: “to reduce social inequalities in children’s health”	yes: “reduce inequalities in child health and development”.	yes: “universally proportionate”, “specific target population”		yes	yes	yes	no	no
Piotrowski CC, Talavera GA, Mayer JA. Healthy Steps: a systematic review of a preventive practice-based model of pediatric care. J Dev Behav Pediatr. 2009 Feb;30(1):91–103.	no	no	no	no	no	no	no	they should be carefully examined to determine if they under-represent known racial and ethnic disparities in the health of new mothers and their infants, thereby potentially biasing the reported outcomes	“need to study the possibility of adapting the programmes according to different sociocultural groups”.”systematic evaluation of potential barriers to successful implementation involving all key stakeholders, including parents”
Van Vuuren C. L. Reijneveld S.A. Van der Wal M. F. Verhoeff A.P. Neighborhood socioeconomic deprivation characteristics in child (0–18 years) health studies: A review. Health & Place, Vol 29, Sep, 2014 pp. 34–42.	“Neighborhood socioeconomic deprivation characteristics”	yes	yes	no, in terms of research methodology	yes	yes	yes	yes	further research is needed to understand the mechanisms that lead to differences in children’s health in relation to the characteristics of neighbourhood deprivation
Gilmer C. Buchan J. L. Letourneau N. Bennett C. T. Shanker S.G.; Fenwick A. Smith-Chant B. Parent education interventions designed to support the transition to parenthood: A realist review. International Journal of Nursing Studies, Vol 59, Jul, 2016 pp. 118–133.	no	no	no	no	no	no	no	no	no
Geens N. Vandenbroeck M. The (ab)sense of a concept of social support in parenting research: a social work perspective. Child & Family Social Work, 19: 491–500. 2014.	no	concentrates on target groups thus limiting a diversified approach			no	no, linked to diversity	in relation to diversity	emphasis on the variety of potential approaches to parenting support, with the “responsibility” not just lying solely with the parents but also shared between state, school and neighbourhood organisations	
Spiteri G. Xuereb R.B. Carrick-Sen D. Kaner E. Martin C.R. Preparation for parenthood: a concept analysis. Journal of Reproductive and Infant Psychology, Vol 32(2), Mar, 2014 pp. 148–165.	no	no	sex, culture and spirituality all influence the concept	no	no, linked to diversity	in relation to diversity	in relation to diversity	parenting is unique to each individual, affected by cultural and societal expectations	no
Halford W. Kim Petch J. Couple Psychoeducation for New Parents: Observed and Potential Effects on Parenting. Clinical Child and Family Psychology Review, Vol 13(2), Jun, 2010 pp. 164–180.	no	no	no	no	no	no	no	an aid specifically for the poorest families that would lead to a reduction in their stress and hence an improvement in the couple’s relationship and consequently their parenting skills	no

#### A fragmented view of the SIH issue in the reviews studied

Only half of the reviews (*n* = 10) addressed the issue of SIH. For the most part, the notions of equity related to their results and conclusions [28, 33, 34, 50, 51]. A fifth (*n* = 4) clearly integrated SIH into their analysis strategy, according to the PRISMA-equity criteria [29, 32, 42, 49].

All of the reviews that tackled the SIH question presented their results and conclusions in relation to equity [21, 29, 32, 42, 44, 49]. Three of these clearly explained their results as supporting a reduction in inequalities [29, 32, 42], and one mentioned an increase in inequalities for some interventions [49]. A return to the original studies that were the subject of these reviews revealed that one programme, Sure Start, presented results that only seemed to benefit the most advantaged socioeconomic gradients [49].

In the studies that examined the link between children’s environment and their health, there was no evidence-based for the socioeconomic characteristics used in the study protocols. However, there was “a general consensus that the combination of income, employment and education is the best way to measure household socioeconomic status” [29].

One review noted that none of the studies directly addressed housing quality as a daily living condition [32]. Very little attention was paid to childcare services in the studies (including only three referenced in Europe [32]).

Relational aspects and social links were also found to have received little research attention even though they increase the feeling of control and help prevent or reduce the risks of depression, stress and anxiety [50]. Research on social links must include a recognition of inequality because the quantity and quality of resources and how they are used can vary enormously depending on the socioeconomic class [50].

The vast majority of studies carried out dealt with at-risk populations, with little attention paid to universal services [28]. Only one review focused on community interventions [42]. Moreover, very few reviews (only three out of the 21 studies) were constructed with the aim of reducing SIH in childhood. Two of these focused on the perinatal period [32, 42], and the third looked at the period from childhood through to adolescence [49].

#### An analysis of the reviews revealed effective strategies for addressing SIH

- There was consensus on the need to support and guide parents during the perinatal period in tackling SIH [28, 32, 42, 49].
- Diversifying approaches shared between state, school and neighbourhood organisations leads to better effectiveness [50]. On the one hand, parenting does not just involve the parents [33, 45]; it also concerns their environment, including the professionals and organisations involved and their social contexts more generally. On the other, parenting is unique to each individual. It is affected by cultural and societal expectations as well as lifestyles [50].
- Proportionate universalism is a solution that was promoted by a number of reviews, most notably those of Morrison et al. (2014) and Welsh et al. (2015) [32, 49]. Michael Marmot (president of the WHO’s Commission on Social Determinants of Health) explained the concept as follows: “To reduce the steepness of the social gradient in health, actions must be universal, but with a scale and intensity that is proportionate to the level of disadvantage. We call this proportionate universalism” [52]. Consequently, there is a need to explore the interventions’ mechanisms to understand which interventions are (in)effective and in what contexts [21, 28, 45].

One review clearly addressed the importance of varying the interventions in accordance with neighbourhood organisations and services [50].

Morrison et al. (2014) showed that, in Europe**,** the most effective interventions (those with the best results and high levels of evidence) were the educational programmes that started at the beginning of pregnancy and included home visits from specialist personnel [32].

## Discussion

To reiterate our main results, the reviews focused, for the most part, on the practices of the professionals. The programmes studied were mainly concerned with improving the mothers’ knowledge and skills. All the reviews that described a proven or promising level of evidence showed that the parenting support programmes increased the mothers’ self-esteem and reduced their anger, anxiety and stress levels and that they improved the infants’ sleep.

Most authors concluded that the quality of the interventions could be improved by developing complementary research examining the interventions’ content, implementation and results. These authors noted that the contexts in which the interventions took place were given little consideration since they were described either only scantly or not at all, making their evaluation difficult.

Only half of the reviews addressed the question of SIH. Most notably, the relational aspects and social links were found to have received little research attention even though they increase the feeling of control and help prevent or reduce the risks of depression, stress, anxiety and other mental illnesses.

### Parenthood and perinatology: Semantic notions

Generally speaking, despite the fact the keywords “parent” and “parenting” were used, the population targeted by the reviews was mainly mothers. Issues relating to fathers or to the couple as a parental entity received little research attention. The methodological difficulty of understanding mother–father–child interactions was often mentioned by authors, although some did refer to the obvious impact of the interrelations of each family member [23, 28, 34, 46].

The notions of parenting and parenting support were found to have multiple meanings and were difficult to define because they depended on many elements. In fact, these notions had different frames of reference depending on the discipline, policy or social history in question [33, 50]. The *Collins English Dictionary* (online) defines “parenthood” as “the state of being a parent”. The definition therefore refers as much to the father as to the mother.

The time immediately before and after a birth, that is from pregnancy to the first months of the child’s life, has received little attention in the studies we have listed. Indeed, most of them focused either on the period preceding the birth or on the infant a few months old [31, 46].

### SIH and perinatal health

#### SIH and vulnerable populations

While the actions, interventions and programmes providing support to parents were described as effective levers in addressing SIH, very few were designed with equity in health as their primary objective.

When authors did address SIH, their reviews mainly targeted population categories such as teenage parents and culturally or economically disadvantaged families. This strategy represents only one reductive vision of SIH and does not, in any way, take into account social gradients. The more socioeconomically disadvantaged an individual’s situation is, the worse their health will be.

This can be observed across the whole social spectrum. Every level in the socioeconomic stratification of the population is affected by SIH [5, 6]. Only one review of all those studied demonstrated the pertinence and effectiveness of a strategy constructed on proportionate universalism [49, 53].

A small number of the reviews (those constructed with equity in health as a clear objective) made reference to an approach that goes beyond the notions of high- or low-risk categorisation according to individual living conditions. One of these examined the value of considering neighbourhood in terms of approximating the notion of socioeconomic deprivation [29].

#### SIH and norms

The fact that the majority of actions were carried out by professionals for the benefit of parents raises the implicit question of a ranking of knowledge [32, 45, 51]. In this sense, traditional types of knowledge, such as group knowledge or that passed on from mother to daughter, tend to be devalued. It is interesting, nevertheless, to note that the notion of childcare emerged as a result of the drive for healthcare control, which was based on a medical discourse that was both injunctive and normative [54]. However, it seems that programmes and actions could be made more effective if they were constructed within a diagnostic approach that is shared between parents, professionals and institutions [28, 31, 34, 49].

The mobilisation of peer groups and the active participation of recipient populations were described as strategies that would increase the effectiveness of actions. The representations of the different stakeholders, whether in terms of their expectations or their needs, still appear to be negligible in programme development. The controversial results from the home-based programmes for socially disadvantaged families [36, 47] could be explained in part by these divergent viewpoints.

### SIH and the social determinants of health

#### Take action early

This review of reviews has identified the most effective actions on health. Therapy groups and telephone support significantly improve parents’ psychosocial health. Psychoeducation programmes that are begun before birth and which allow parents the opportunity to actively participate are the most effective [29, 41].

#### Act with the families

Facilitators or difficulties in setting up the actions were never described. This review of reviews has also revealed, in particular, an assumed lack of knowledge among parents and even a lack of analysis of the relational modalities between the professionals and the parents [21, 31, 35, 45, 48]. Collaboration involves the need for a common language between professionals from different disciplines, parents with multiple issues with different objectives. This review has shown, however, that studies focusing on the ability to listen and the quality of the professionals–mothers–parents–newborns relationship are under-represented [35, 44]. Highlighting and theorising these interrelations represents an approach to understanding how interventions function, which enables them to be made more effective [55–57].

#### Act in interaction with the context

The ability of each person to take action to improve their health depends much on the social context in which they live, think and work. Health promotion does not just concern health services but also the social determinants of health that make up this context [54–56]. Social determinants have been widely shown to have a strong influence on health [2, 5, 6, 53].

Proposing programmes and actions to tackle SIH therefore calls for a global, dynamic approach to its social determinants. An analysis of the relationships between the social, economic, cultural and environmental contexts in which these projects are rolled out would allow us to understand the functioning of these interventions.

#### Theorise to act

Almost all the reviews highlighted the lack of description given to the intervention development processes. A number of authors noted that the interventions were evaluated on their effectiveness but that insufficient information had been given about the interventions’ settings or their populations [28, 34, 44]. This lack of contextual elements raises the question of the transferability of the programmes or actions described in the reviews analysed. For example, many programmes focused on parents’ knowledge and skills but neglected to first define the specific needs of the populations targeted. It is therefore difficult to demonstrate their effectiveness and to envisage their adaptation to other contexts.

### Limitations of this review

While many public health journals value publications from the human and social sciences, this synthesis of knowledge was carried out using only bibliographic health databases. As a result, it has undoubtedly overlooked certain elements within the sociological approaches.

The methodological choice to synthesise knowledge using articles from scientific journals precluded us from analysing public health policies or the social policies that these programmes and actions were interacting with.

It should be noted that the reviews we studied were written in English, which could have led to a possible selection bias. If this was the case, the bias remained limited since the majority of scientific publications are written in English.

Because the reviews focused on high-income countries, the results of this study cannot be extrapolated to low income countries, where the situations may be different.

## Conclusions

This focus on the current knowledge concerning action on social inequalities in perinatal health shows that the approach remains both modest and reductive. On the one hand, very few authors have considered the notion of equity in health, and, on the other, the vision of SIH remains limited. Parental programmes focused, for the most part, only on the mothers, and the actions tended to target the most disadvantaged populations, with no consideration of the social gradients of health.

This review shows that the majority of the publications came from English-speaking countries. Currently, to our knowledge, no study has been carried out in Europe on parenting support as a means of addressing SIH among mothers and their newborns.

Parenting support interventions are complex. Their effects are variable, and they result from multiple actions, with the different stakeholders interacting in and with a particular dynamic environment. In future research, the methodological challenge will be to understand how, for whom and in what conditions interventions function.

## Abbreviations

AMSTAR: Assessing the Methodological Quality of Systematic Reviews
MeSH: Medical Subject Heading
PRISMA: Preferred Reporting Items for Systematic Reviews and Meta-Analyses
SIH: Social inequalities in health
WHO: World Health Organization
